# C-Reactive Protein in Atherosclerosis—More than a Biomarker, but not Just a Culprit

**DOI:** 10.31083/j.rcm2410297

**Published:** 2023-10-19

**Authors:** Kürsat Kirkgöz

**Affiliations:** ^1^University Medical Center Hamburg-Eppendorf, 20251 Hamburg, Germany

**Keywords:** C-reactive protein, atherosclerosis, biomarker, inflammation, mCRP, pCRP, cardiovascular disease, thrombosis

## Abstract

C-reactive protein (CRP) is a pentraxin that is mainly synthesized in the liver in response to inflammatory cytokines. It exists in two functionally and structurally distinct isoforms. The first is a highly pro-inflammatory and mostly tissue-bound monomeric isoform (mCRP). The second is circulating pentameric CRP (pCRP), which also serves as a substrate for the formation of mCRP. CRP is elevated during inflammatory conditions and is associated with a higher risk of cardiovascular disease. The aim of this review is to examine the current state of knowledge regarding the role of these two distinct CRP isoforms on atherogenesis. This should allow further evaluation of CRP as a potential therapeutic target for atherosclerosis. While it seems clear that CRP should be used as a therapeutic target for atherosclerosis and cardiovascular disease, questions remain about how this can be achieved. Current data suggests that CRP is more than just a biomarker of atherosclerosis and cardiovascular disease. Indeed, recent evidence shows that mCRP in particular is strongly atherogenic, whereas pCRP may be partially protective against atherogenesis. Thus, further investigation is needed to determine how the two CRP isoforms contribute to atherogenesis and the development of cardiovascular disease.

## 1. Introduction 

C-reactive protein (CRP) is a pentraxin that is mainly synthesized in the liver in response to inflammatory cytokines, especially interleukin (IL)-1, IL-6 and tumor necrosis factor (TNF)-alpha [1, 2]. CRP can also be synthesized in response to local inflammatory cytokines by human coronary artery smooth muscle cells (HCASMCs) [2], human coronary artery endothelial cells (HCAECs) [3], and in diseased coronary artery venous bypass grafts [4, 5]. It is first synthesized as monomers and then assembled into the pentameric form in the endoplasmic reticulum of cells [1, 2]. Native CRP circulates in the soluble pentameric form (pCRP), and its core physiological function can be summarized as opsonization. This is mediated by its ability to interact with Fc receptors on phagocytic cells and activate complement through ${\mathrm{Ca}}^{2+}$-dependent interactions with phosphocholine. The latter is distributed on cell membranes and is associated with pathogenic lipopolysaccharides [6, 7]. pCRP can be measured using a high-sensitivity assay (hsCRP) [7]. Another functionally and structurally distinct isoform of CRP also exists in the form of highly pro-inflammatory monomeric CRP (mCRP). This is formed at local inflammation sites as a consequence of pCRP dissociation. In contrast to pCRP, mCRP is mostly tissue bound and has significantly lower aqueous solubility [8, 9]. There are also structures consisting of partially dissociated pCRP existing as membrane-bound intermediates (described as mCRP m or pCRP*), which act similar to mCRP. Many reports have recently been published that suggest an active role for mCRP in atherogenesis.

CRP is an acute-phase protein that is elevated in all inflammatory diseases and is associated with a higher risk of cardiovascular disease [10, 11, 12]. CRP levels can increase during immune reactions to 5–10 mg/L in mild cases and up to 320–550 mg/L in the most severe cases [13, 14], but are usually <5 mg/L in atherosclerosis. The hsCRP assay has a threshold of just 0.28 mg/L and can thus be used to measure CRP in the low range. Cardiovascular risk scores such as the Reynolds score include the classical risk factors as well as the hsCRP level, and these correlate directly with major adverse cardiovascular events (MACE) such as myocardial infarction (MI) [11]. Recent clinical trials and experimental research have shown that CRP could be a mediator for atherosclerosis as well as a biomarker [12]. Recent therapeutic trials have targeted CRP, and additional mechanisms that implicate CRP in atherogenesis are still being identified [12]. *In vitro *experiments have reported that CRP preparations were contaminated by bacterial products or other factors [15, 16]. Another study found no inflammation after purified CRP from pooled normal donor plasma was infused into 7 healthy adult human volunteers, but this study did not distinguish between mCRP and pCRP [17]. Therefore, it remains to be determined whether CRP is a proatherogenic mediator as well as a biomarker. The aim of this review is therefore to examine the current state of knowledge regarding the effect of distinct CRP isoforms on atherogenesis. This should facilitate the evaluation of CRP as a potential therapeutic target for atherosclerosis, as well as clarifying some of the current controversies.

Atherosclerosis is the leading cause of ischemic diseases such as MI, stroke, and peripheral artery disease (PAD). Besides the hsCRP level, there are many known risk factors for atherosclerosis, including hypercholesterolemia, hypertension, diabetes and smoking [11, 18]. Lipid-lowering and antithrombotic drugs such as statins and acetylsalicylic acid are the most successful way to prevent and treat atherosclerosis. Despite these advances, questions remain regarding the pathogenesis of atherosclerosis. Furthermore, novel therapeutics are needed for patients that cannot be adequately treated using current drugs [18]. Atherosclerosis is a progressive inflammatory disease, with the inflammatory process caused by continuous exposure to pathogenic factors such as hypertension, stress and smoking that damage the arterial intima. The resulting dysfunction and permeability of the endothelium leads to further infiltration of low-density lipoprotein (LDL) cholesterol into the extracellular matrix, where it becomes a target for oxidative and enzymatic modification. This triggers a series of pro-inflammatory reactions, leading to enhanced diapedesis of immune cells into the subendothelial tissue, remodeling of the vascular smooth muscle cells (VSMC), and accumulation of LDL cholesterol and calcium in the vessel wall. Following formation of the fatty streak and lipid accumulation, the resident macrophages and VSMC-derived macrophage-like cells take up LDL and become loaded with lipids, giving them a foamy appearance. These foam cells secrete various substances involved in plaque growth, causing the arterial lumen to narrow and constricting blood flow. Many of the foam cells undergo necrosis and are partially removed by macrophages, leading to the release of lipids and matrix metalloproteinases (MMPs). MMPs degrade the extracellular matrix, thus making the plaque susceptible to rupture. With time, the plaque becomes unstable and ruptures, leading to thrombus formation that can completely block blood perfusion [18, 19]. Atherogenesis can therefore be divided into the following stages: (1), endothelial dysfunction and initial oxidized LDL (oxLDL) uptake; (2), migration of leukocytes and smooth muscle cells into the vessel wall; (3), foam cell formation and degradation of extracellular matrix; and (4), plaque rupture and thrombosis. Depending on its isoform, CRP may have pathogenic or beneficial effects in each of these stages. For the remainder of this review, the CRP abbreviation is used when describing studies in which no distinction was made between pCRP and mCRP.

## 2. Endothelial Dysfunction and Initial oxLDL Uptake

Nitric oxide (NO) produced by endothelial cells is an essential regulator of vascular homeostasis. Endothelial dysfunction is characterized by decreased sensitivity to NO and reduced production capacity for NO. This ultimately causes an imbalance in vascular homeostasis, leading to a prothrombotic, proinflammatory and less compliant blood vessel wall that is manifested clinically as hypertension [20]. NO plays an essential role in endothelial function by promoting the relaxation of VSMCs to cause vasodilation. NO is also involved in regulating platelet and leukocyte adhesion, thrombosis, and fibrinolysis [21]. However, decreased NO production is associated with pathogenic atherosclerosis events. Elevated CRP concentrations decrease the release of NO and reduce bioactivity in human endothelial cell cultures [22]. Single intravenous injection of CRP in a mouse model contributed to endothelial dysfunction and hypertension by inhibiting NO release [23]. CRP also inhibits NO-linked angiogenesis, which is a crucial mechanism in chronic ischemia [22]. In a CRP overexpressing (hCRPtg) mouse model, both the release of NO and the expression of phosphorylated endothelial NO-Synthase (eNOS) were significantly reduced in isolated aortic segments [24]. However, in all of these studies in which CRP was either injected or overexpressed in its pentameric form, its exact isoform was not verified, i.e., by specific antibodies that recognize the isoforms [25]. Hence, it is not known whether the observed effect was caused by pCRP or mCRP. The latter is produced gradually by the dissociation of pCRP at membrane sites [26]. Furthermore, prolonged storage of purified CRP in solutions that lack calcium results in the spontaneous dissociation of pCRP to mCRP. Indeed, in contrast to more recent publications, the results of early work on CRP can seem unclear and contradictory. The earlier work often did not specify which CRP isoform was measured or used in experiments, and hence the responses attributed to pCRP might actually be due to dissociated mCRP. Further work is therefore needed to determine which CRP isoform is responsible for the observed effects regarding NO dynamics. The existence of the two CRP isoforms is now well established and several common techniques and specific antibodies are available to distinguish the isoforms. Some of the early studies also tried to distinguish between the isoforms. One study showed that treatment with pCRP, but not mCRP, downregulated eNOS and thus impaired endothelial function in ApoE knockout mice via increased inducible NO-Synthase (iNOS) activity [27]. However, the observed effect of pCRP in this work could also be due to its dissociation into mCRP at the respective membrane sites, while the administered mCRP may have shown no significant effect due to its low plasma solubility and high sensitivity to proteolysis [28].

The effect of the vasodilator prostacyclin was attenuated in CRP-treated human aortic endothelial cells [29]. However, it is unclear whether CRP has a positive effect on the natural NO counterpart endothelin-1 (ET-1) [30], or whether the activity of the vasoconstrictor ET-1 is not affected by increased CRP [31]. Since these early studies did not distinguish between the two CRP isoforms, such controversies may be clarified by further research with proper consideration of the distinct isoforms. In contrast to CRP, it is assumed that ET-1 induces the release of prostacyclin [32, 33].

Lectin-like oxidized LDL receptor-1 (LOX-1) is an endothelial receptor for oxLDL with a critical impact on oxLDL-induced endothelial dysfunction. The expression of LOX-1 was increased following incubation of human aortic endothelial cells with 25 µg/mL pCRP for 24 hours or with 5 µg/mL pCRP for 7 days [34]. Importantly, CRP-induced endothelial LOX-1 expression was dependent on the presence of ET-1 [34]. Given that CRP acts here at membrane sites for hours, the pentameric CRP may have dissociated into mCRP. Therefore, it can be speculated that mCRP caused the increase in LOX-1 expression. However, the underlying mechanism involving CRP in the development of atherosclerosis via its influence on endothelial function requires further investigation, with a special focus on the two distinct isoforms.

In patients with hypertension, a positive correlation was reported between the levels of circulating hsCRP and pulse wave velocity, which is a functional indicator of arterial stiffness and endothelial dysfunction [35, 36]. This correlation may be due to direct involvement of CRP in the development of arterial stiffness via an enhanced vascular response to angiotensin II and aldosterone, thus supporting the notion that CRP is a mediator of endothelial dysfunction [37, 38].

Other studies also support a direct role for CRP in the development and/or progression of atherosclerosis via the blocking of endothelial regeneration [39, 40, 41, 42]. In this regard, a negative correlation was reported between circulating hsCRP plasma levels and endothelial progenitor cells (EPCs), which play a crucial role in endothelial regeneration [39]. Using a rat model, it was shown that CRP impairs EPC antioxidant defense by upregulating expression of the receptor for advanced glycation end products (RAGE), thereby increasing the sensitivity of EPC towards oxidative stress-mediated apoptosis [40]. Moreover, high CRP concentrations have been shown to facilitate telomerase inactivation in EPCs isolated from peripheral venous blood [41]. More recent studies have shown that mCRP, but not pCRP, can promote injury and apoptosis of human cultured HCAECs via phosphorylation and activation of p38 mitogen-activated protein kinase (p38 MAPK) [42].

**Fig. 1.**
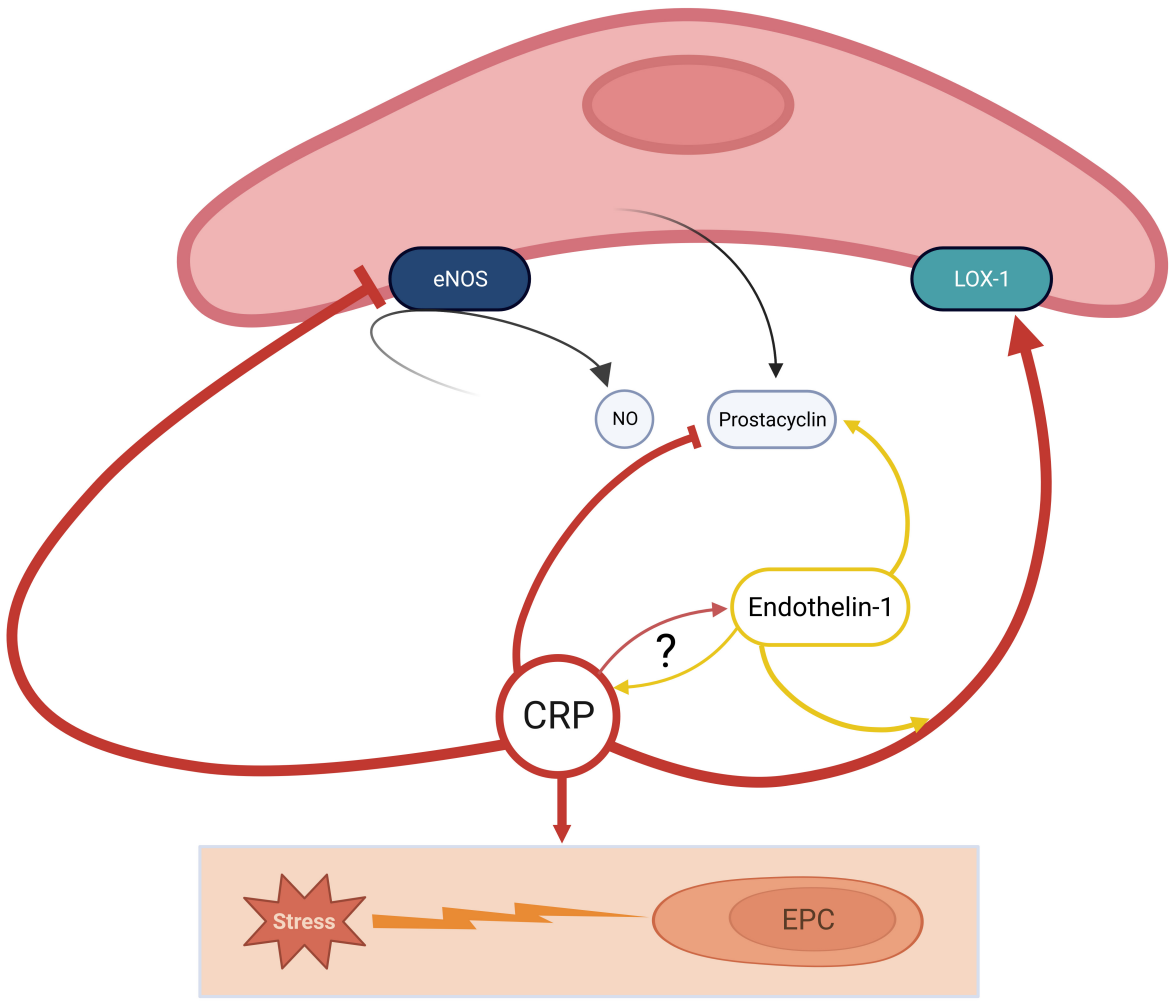
**CRP actively participates in the development of a pro-atherosclerotic environment**. CRP interacts with eNOS to inhibit the production of NO and directly inhibits prostacyclin activity, which is contrary facilitated by Endothelin-1. Furthermore, CRP stimulates LOX-1 in presence of Endothelin-1; Finally, CRP promotes apoptosis of EPCs by oxidative stress; there are controversial findings about the direct relation of Endothelin-1 and CRP yet. Little is known about the different activities of pCRP and mCRP. CRP, C-reactive Protein; eNOS, endothelial NO-synthase; LOX-1, lectin-like oxidized LDL receptor-1; EPC, endothelial progenitor cell; NO, nitric oxide; mCRP, monomeric C-reactive protein; pCRP, pentameric C-reactive protein; LDL, low-density lipoprotein.

In summary, it appears that CRP impairs normal endothelial function and regeneration capacity, thereby leading to endothelial dysfunction and the initiation of atherosclerosis (Fig. 1). However, the role of each CRP isoform in the early stages of atherogenesis is still largely not known, nor is it clear which isoform facilitates telomerase inactivation in EPCs from peripheral venous blood [29].

## 3. Migration of Leukocytes and Smooth Muscle Cells into the Vessel Wall

A series of inflammatory responses begins in the intima following damage to the endothelium and subsequent oxLDL uptake, leading to increased activation and diapedesis of immune cells into the subendothelial tissue. Driven by the lipid accumulation, monocytes migrate and myocardin expression in VSMCs is silenced, thereby causing a phenotypic switch to macrophage- and myofibroblast-like cells and stabilizing the plaque [43].

**Fig. 2.**
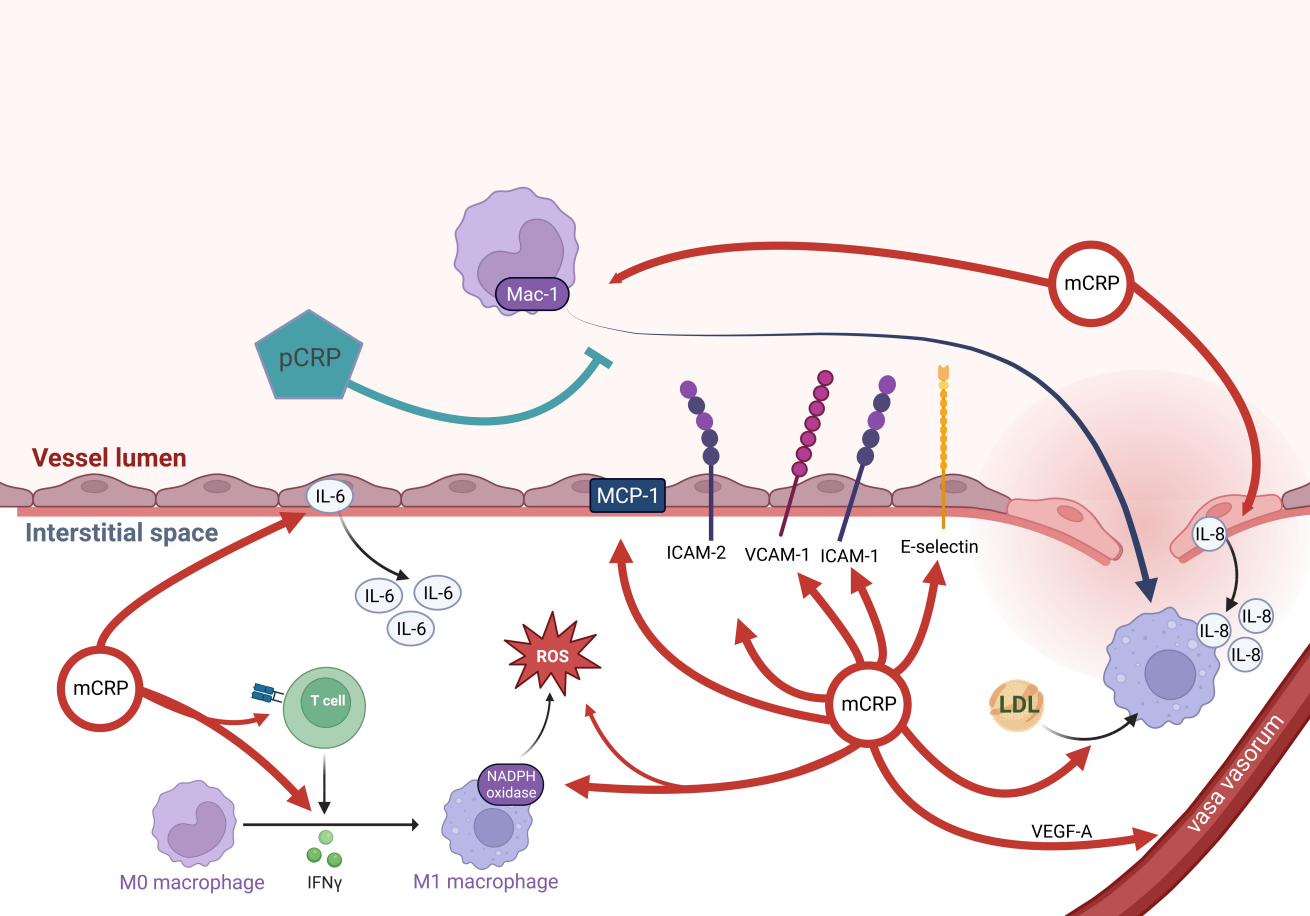
**mCRP mediates an inflammatory response in the intima**. Monocytes are immigrating. Via various mechanisms, mCRP facilitates diapedesis, chemotaxis, and activation of monocytes, while pCRP inhibits monocyte adhesion. Finally, (ox)LDL uptake is promoted by mCRP. mCRP can trigger CD4+ T cell effector responses, leading to IFN-γ production. mCRP can directly polarize the macrophages into the pro-inflammatory M1 phenotype. Growth of VV is enhanced by elevated VEGF-A expression via mCRP. Finally, mCRP directly acts pro-inflammatory by inducing ROS and IL-6 production. m/pCRP, monomeric/pentameric C-reactive Protein; Mac-1, Macrophage-1 antigen; ROS, reactive oxygen species; VCAM-1, vascular cell adhesion molecule-1; ICAM-1/2, intercellular adhesion molecule-1/2; MCP-1, monocyte chemoattractant protein-1; mCRP, monomeric C-reactive protein; CD, cluster of differentiation; LDL, low-density lipoprotein; IFN, interferon; VV, vasa vasorum; VEGF-A, vascular endothelial growth factor-A; IL, interleukin.

CRP plays an essential role in the activation and diapedesis of leukocytes into the subendothelial tissue (Fig. 2). Incubation of monocytes with various concentrations of mCRP, but not pCRP, results in a dose-dependent increase in activation of the integrin Mac-1 at the cell surface. This is crucial for cellular events such as rolling, adhesion, and transmigration (i.e., by binding to intercellular cell adhesion molecule-1, ICAM-1) [25]. Interestingly, monocyte adhesion induced by mCRP can be partially inhibited by pCRP, as well as by blocking cluster of differentiation (CD)64, CD32 and CD16 [25]. Incubation of HCAECs with mCRP, but not pCRP, resulted in time- and dose-dependent increases in the cell-surface expression of vascular cell adhesion molecule-1 (VCAM-1), ICAM-1, ICAM-2, and E-selectin via the activation of p38 MAPK [42, 44], thus promoting the activation and diapedesis of leucocytes. Furthermore, mCRP induced time- and dose-dependent increases in IL-8 mRNA and protein levels *in vitro*, driven by the upregulation of NF-kappa B [45]. IL-8 is known to have chemotactic effects on monocytes and granulocytes. Additionally, mCRP induced time- and dose-dependent increases in the expression of monocyte chemoattractant protein-1 (MCP-1) and the production of IL-6 *in vitro * [44, 45]. This leads to an enhanced inflammatory response by MCP-1, recruitment of monocytes and IL-6-activated lymphocytes, and subsequent induction of CRP gene expression [44, 46]. Conversely, prolonged culture with pCRP (>1 day) was needed to detect cell activation, suggesting the potential dissociation of pCRP into mCRP. Interestingly, mCRP has been shown to mediate monocyte infiltration into damaged tissues in a rat model of renal ischemia/reperfusion injury [47] and in a murine model of MI [48]. *In vivo *and *in vitro* studies also showed that mCRP could polarize macrophages into the pro-inflammatory M1 phenotype by activating the JNK signaling pathway [48]. M1 macrophages produce cytokines such as TNF-α, IL-6 and IL-1β, which in turn facilitate the production of CRP [1, 2, 46]. This reveals a positive feedback mechanism for CRP. Therefore, mCRP appears to facilitate the chemotaxis, diapedesis, and migration of antigen-presenting cells and to consolidate local inflammation in the vessel wall, whereas pCRP is able to partially reverse this process.

CRP has been shown to upregulate expression of the Angiotensin II receptor AT1-R and increase the number of AT1-R binding sites in cultured human VSMCs, thereby facilitating Ang II–induced reactive oxygen species (ROS) production, VSMC migration, proliferation, and vascular remodeling [38, 49]. Another study confirmed the direct effect of CRP on ROS production *in vivo * [50]. Earlier studies on the interaction between ROS and CRP did not distinguish the CRP isoforms. However, more recent studies showed that mCRP, but not pCRP, could increase ROS production via the NADPH oxidase enzyme complex in macrophages [44, 47].

Another important mechanism in intimal inflammation involves the vasa vasorum (VV), which are known to correlate strongly with the progression of atherosclerosis in human coronary arteries [51]. VV facilitate the infiltration of inflammatory cells, intimal thickening, intraplaque hemorrhage, and subsequent atherothrombosis leading to ischemia [52]. mCRP, but not pCRP, significantly promoted vascular endothelial growth factor-A (VEGF-A)-induced neovascularization via the PI3K/Akt and MAPK/ERK signaling pathways. This provides new evidence that CRP promotes the growth of VV in atherosclerosis [53, 54, 55, 56]. On the other hand, CRP can also have deleterious effects on EPCs by decreasing their survival, inducing apoptosis, and impairing their differentiation [39, 40, 41]. Although this may seem controversial, the deleterious effects are assumed to be caused by pCRP and not mCRP [57]. The anti-angiogenic effects may be caused by pCRP, which circulates in the blood and may have anti-angiogenic effects on the whole system. In contrast, the angiogenic effect of mCRP is only observed locally near the inflamed tissue. Further research should focus on the mechanisms by which the distinct CRP isoforms can hinder or induce angiogenesis.

Finally, recent studies have shown that mCRP can trigger CD4+ T cell effector responses in the absence of antigens by inducing spontaneous signaling of the T cell receptor (TCR). mCRP binds cholesterol in the plasma membranes of CD4+ T cells, thereby shifting the conformational equilibrium of TCR to the cholesterol-unbound, primed state [58]. While this favors the transformation of innate immune recognition by CRP into immediate adaptive immune responses, there is also the potential for autoimmune activity in atherosclerosis. Spontaneous signaling by the TCR leads to release of interferon (IFN)-γ, which is known to be expressed at high levels in atherosclerotic lesions [59]. IFN-γ is the classic macrophage-activating factor. It induces the expression of genes that regulate lipid uptake and is a key trigger in the formation and release of ROS. IFN-γ also has important effects on endothelial cells by promoting the expression of adhesion molecules. Atherogenic effects of IFN-γ have also been shown in murine models [59].

## 4. Foam Cell Formation and the Degradation of Extracellular Matrix

Following formation of the fatty streak and subsequent lipid accumulation, macrophages and VSMC-derived macrophage-like cells take up LDL and become loaded with lipids, giving them a foamy appearance. These cells then secrete various substances involved in plaque growth, and their necrosis also promotes inflammation, thereby contributing to cardiovascular disease [60, 61, 62, 63, 64]. Various studies have been published on the effect of CRP on foam cell formation. *In vitro* and *in vivo* studies have shown that mCRP facilitates oxLDL uptake by macrophages [60, 65], while other studies have presented evidence that CRP may increase native LDL uptake by macrophages [62]. It has also been suggested that macrophages take up CRP-LDL complexes [66]. Interestingly, the binding of pCRP to modified LDL prevented LDL binding to monocytes* in vitro*, whereas mCRP binding to modified LDL enhanced its interaction with monocytes [67]. Modified LDL is atherogenic because it is recognized by monocytes, resulting in LDL-loaded foam cells [68]. Another study showed that binding of mCRP to LDL correlates with the amount of non-esterified cholesterol in LDL [69]. Besides LDL, macrophages can phagocytose single cholesterol crystals via CRP-dependent mechanisms [70]. This may be atheroprotective in the early stages because it could help to clear modified self-structures, but the intracellular accumulation could also promote foam cell formation. In this regard, *in vitro* studies have shown that CRP reduces cholesterol efflux from human macrophage-derived foam cells by silencing the expression of ATP-binding cassette transporter A1 (ABCA1) and ATP-binding cassette transporter G1 (ABCG1), which are essential molecules in mediating this efflux [71].

However, there are also controversial findings which support the notion that CRP does not mediate foam cell formation and that CRP has anti-atherosclerotic functions. By binding to enzymatically modified LDL at the same binding site as phosphocholine, CRP could prevent the uptake of LDL by macrophages and hence the formation of foam cells [72, 73], as well as suppressing the pro-inflammatory and oxidative activities of macrophages [73]. Additionally, CRP has been shown to inhibit the further oxidation of ox-LDL [74, 75]. Moreover, *in vitro* experiments have shown that mCRP decreases the uptake of acetylated LDL by human umbilical vein endothelial cells (HUVECs) [76]. Nevertheless, some of the studies that investigated the effects of CRP on LDL uptake and foam cell formation did not distinguish between the different isoforms of CRP, even though the bioactivity of CRP is known to depend on its structural status [7, 8, 9]. A few studies have purposely distinguished between the pCRP and mCRP isoforms, but these have also reported somewhat controversial findings [60, 65, 76]. A possible explanation for the discrepancies could be a mutant mCRP that was atheroprotective in a murine model of atherosclerosis. When bound to LDL, this mutant mCRP could reduce foam cell formation and local inflammation [77]. Further studies on the different CRP isoforms are required to clarify the above controversies, and in particular their interaction with LDL.

## 5. Plaque Rupture and Thrombosis

Many foam cells in the thickened intima undergo necrosis and are partially removed by macrophages, leading to the release of lipids and MMPs. The MMPs degrade the extracellular matrix, including the collagen and elastin in the overlying fibrous cap, thereby rendering the plaque susceptible to rupture.

CRP may contribute to plaque instability since it has been shown *in vitro* to induce the expression and collagenase activity of MMP-1, -2, -9 and -10 [78, 79, 80, 81]. Recent studies suggest that CRP induces MMP expression via the phosphorylation and activation of ERK1/2 and p38 MAPK [82]. Given that mCRP, but not pCRP, can phosphorylate and activate p38 MAPK and ERK [42, 53, 54, 55, 56], increased MMP expression is likely to be caused by mCRP rather than pCRP.

The growth of intraplaque neovessels originating from VV is promoted by CRP during atherosclerosis. These vessels can be immature and hence predisposed to leakage and are therefore regarded as the primal cause of intraplaque hemorrhage [51, 52, 53, 54, 55, 56].

CRP also facilitates thrombosis by mediating coagulation, with mCRP markedly increasing the expression and activity of tissue factor (TF) in cultured endothelial cells [83]. HUVECs incubated with mCRP, but not pCRP, showed faster fibrin polymerization and a significantly increased density of fibrin clots [83]. This suggests a mechanism by which mCRP promotes pathological fibrin formation after vascular leakage. Furthermore, CRP has been shown to increase TF expression and activity in monocytes and VSMCs, both *in vitro* and *in vivo* [84, 85, 86]. Infused CRP has been shown to activate coagulation in humans by increasing circulating levels of von Willebrand factor, prothrombin F1+2, and plasminogen activator inhibitor type-1 (an inhibitor of t-PA), thus impairing the degradation of fibrin clots [87].

**Fig. 3.**
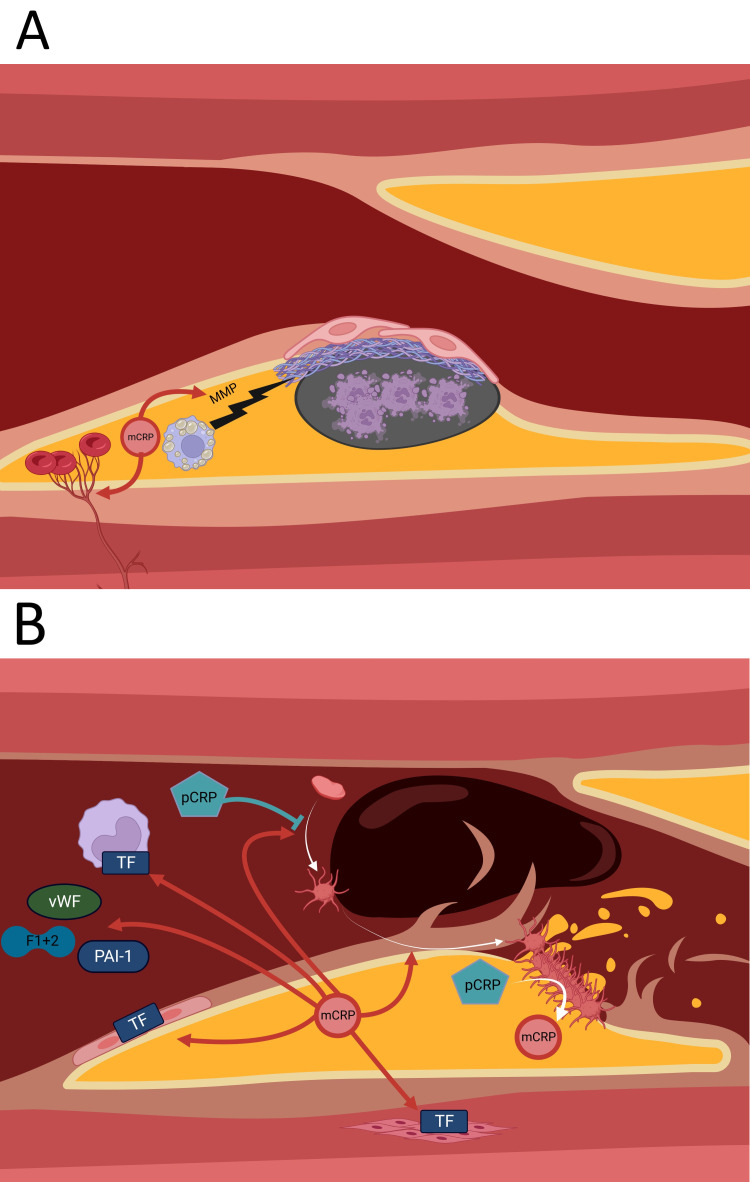
**Some of the pro-thrombotic effects of mCRP**. (A) mCRP facilitates degradation of the fibrous cap and thus finally plaque rupture via inducing expression of MMPs by foam cells. mCRP mediates growth of VV into the plaque, which become leaky and cause intraplaque hemorrhage. (B) mCRP induces TF expression in endothelial cells, monocytes and VSMCs. Circulating CRP increases vWF, PAI-1 and prothrombin F1+2 levels. Also, mCRP promotes platelet activation and adhesion, while pCRP inhibits platelet activation. Activated platelets can dissociate pCRP into mCRP. MMP, matrix metalloproteinase; m/pCRP, monomeric/pentameric C-reactive Protein; vWF, von-Willebrand factor; TF, tissue factor; PAI-1, plasminogen activator inhibitor type 1; F1+2, prothrombin fragment 1+2; VV, vasa vasorum; VSMCs, vascular smooth muscle cells.

Furthermore, mCRP but not pCRP was able to facilitate thrombosis *in vitro* by inducing platelet activation and deposition [88], platelet adhesion to ECs and monocytes via the upregulation of P-selectin [89, 90, 91], and thrombus growth [89, 92]. However, pCRP inhibits platelet activation and hinders the binding of platelets to neutrophils [93]. Interestingly, there appears to be bidirectional CRP-platelet crosstalk, with CRP affecting platelets, but platelets also able to change the conformational status and bioactivity of CRP. Thus, activated platelets have been shown to convert pCRP to mCRP [25]. Antibody (abciximab) blocking of the glycoprotein IIb/IIIa on activated platelets prevents the dissociation of pCRP to mCRP and reduces platelet deposition at the arterial wall [88]. In summary, mCRP has pro-thrombotic action, while pCRP has weak anti-thrombotic effects (Fig. 3).

## 6. Targeting of CRP in Atherosclerosis Therapy

As recommended by current guidelines on the primary prevention of cardiovascular disease (CVD), the most important way to prevent atherosclerotic vascular disease is to promote a healthy lifestyle throughout life [94]. Indeed, both aerobic and resistance exercise have been shown to decrease CRP levels independently of statin therapy [95]. Two meta-analyses have reported that decreases in body mass index (BMI) and body fat correlate with decreased CRP levels [96, 97]. Besides body-weight reduction and regular physical activity, the cessation of smoking has also been shown to decrease CRP levels [98]. In addition, diet has a significant impact on subclinical vascular inflammation. Several dietary factors have been shown to decrease CRP levels, including fiber-rich foods, whole grains, fruits (especially berries), omega-3 fatty acids, antioxidant vitamins C and E, and certain trace minerals such as zinc [95]. These foods may also help to reduce the pro-inflammatory postprandial state, which is especially evident after the ingestion of meals that are high in saturated fat [95]. Current American Heart Association (AHA) guidelines on the primary prevention of CVD recommend a healthy diet that emphasizes the intake of vegetables, fruit, nuts, lean vegetable or animal protein, whole grains, and fish, while minimizing the intake of trans fats and red meat [94].

According to current guidelines, statin therapy is the first-line treatment for primary prevention of atherosclerotic CVD [94]. Statins are cholesterol-lowering drugs that act by inhibiting 3-hydroxy-3-methylglutaryl coenzyme A (HMG-CoA)-Reductase, an essential enzyme for cholesterol biosynthesis. The CRP-lowering effect of statins may be mediated by NO, since statins are known to promote its production [99, 100]. Early discussion regarding the targeting of CRP began with a clinical trial of statin therapy in CVD (JUPITER study, Justification for the Use of Statins in Prevention: An Intervention Trial Evaluating Rosuvastatin) [101]. A total of 17,802 participants (men >50 years, women >60 years) with LDL cholesterol levels <130 mg/dL, hsCRP levels >2 mg/L, and no history of diabetes or CVD were randomized to receive 20 mg rosuvastatin daily or placebo. The JUPITER trial found a significantly reduced incidence of MACE in apparently healthy individuals with normal lipid levels but elevated hsCRP levels. This result suggested that CRP was a potential target for CVD therapy [101] and supported the findings of various experimental studies that attributed atherogenic effects to CRP. Another important trial was IMPROVE-IT (Improved Reduction of Outcomes: Vytorin Efficacy International Trial), which compared dual treatment with ezetimibe, a blocker of the gastrointestinal cholesterol transporter NPC1L1, and simvastatin versus simvastatin alone [102]. This trial confirmed that reduction of hsCRP to <2.0 mg/L decreased the rate of MACE by 28–33%, which was comparable to patients with an LDL level of <1.8 mmol/L. However, ezetimibe monotherapy [103] and other lipid-lowering drugs such as PCSK9 inhibitors had no significant effect on hsCRP levels [104].

Currently, there are no therapies that specifically target CRP. Targeting the expression of CRP or directly targeting circulating pCRP would likely affect its important physiological function as an acute-phase protein and potentially impair antibacterial defenses [6, 7, 8], making this approach unsuitable [105]. As described above, pCRP may also have some atheroprotective functions by reducing monocyte adhesion or inhibiting platelet activation [25, 93]. However, the experimental findings presented above suggest that a novel therapeutic approach may be to block the conversion of pCRP to mCRP, since mCRP is the proatherogenic and prothrombotic agent. An early approach to inhibit the dissociation of pCRP to mCRP was the use of bivalent compounds such as 1,6-bis phosphocholine-hexane (1,6-bisPC). This drug bridges the phosphatidylcholine-binding sites of two separate pCRP molecules, thereby bringing the phosphatidylcholine-binding surfaces together and concealing them. The decameric structure formed by this action prevents conformational changes in pCRP by blocking the binding of other ligands to the phosphatidylcholine-binding sites. In a rat model of induced MI, 1,6-bisPC treatment decreased the infarct size and cardiac dysfunction induced by CRP injection [106]. Treatment with 1,6-bisPC also hindered mCRP deposition and reduced leukocyte infiltration into the damaged myocardium in a rat model of ischemia/reperfusion injury [47]. Although this method could affect physiological pCRP function by cross-linking two pCRP disks, 1,6-bisPC has a suboptimal pharmacokinetic profile [106]. More recent approaches have used a novel phosphatidylcholine-mimetic compound, C10M [3-(dibutyl amino) propylphosphonic acid]. This agent binds to the phosphatidylcholine-binding site on pCRP and competitively inhibits it from binding to exposed phosphatidylcholine head groups on bioactive lipids, such as in cell membranes, thereby preventing the formation of mCRP [107]. C10M-bound pCRP is still accessible to other interacting ligands, so the physiological function of pCRP (i.e., antibacterial defense) remains unimpaired [105, 107]. *In vitro* studies have shown that C10M decreases pCRP binding to activated platelets, mCRP-induced expression of ICAM-1 and VCAM-1, ROS generation, and leucocyte adhesion. In a rat model of CRP-induced renal ischemia/reperfusion injury, C10M was found to abrogate mCRP deposition and monocyte accumulation in affected organs, reduce mCRP-driven exacerbation, and improve renal excretory function. Moreover, C10M has been shown to prevent CRP-mediated allograft rejection in a rat model of hindlimb transplantation [107]. However, the bioavailability of C10M is <30%. No phase-I or phase-II metabolism was seen, but rapid elimination via urine excretion was observed [108]. C10M may therefore need further modification of its present form in order to improve the pharmacokinetic profile. Overall, this approach opens a promising and novel therapeutic path to treat atherosclerotic diseases.

Direct targeting of mCRP remains a possibility, but has yet to be studied thoroughly. A monoclonal antibody against mCRP was found to reduce rheumatoid arthritis symptoms, joint inflammation, pannus formation and bone destruction in a mouse model of arthritis [109]. This antibody also attenuated glomerular damage and the progression of proteinuria in a mouse model of lupus nephritis [109]. An anti-mCRP antibody completely blocked mCRP-induced chronic memory loss in a murine model of dementia in which mCRP was stereotactically injected into the hippocampus to produce symptoms of neurodegeneration [110]. Further research is needed to investigate this targeted approach for the treatment of atherosclerosis.

## 7. Conclusions

In conclusion, the majority of evidence indicates that mCRP is the proatherogenic CRP isoform, while pCRP is either neutral or has mild anti-atherogenic effects. Therefore, it seems clear that CRP should be used as a therapeutic target for atherosclerosis and CVD. The current evidence indicates that CRP is more than an innocent bystander in atherosclerosis, and not just a “culprit”. This knowledge could be used to develop novel therapeutics that specifically block the atherogenic effects of mCRP, without blocking the various physiological functions of pCRP. However, there are still gaps in our knowledge about the effects of the two different CRP isoforms on atherogenesis. Controversies that still require clarification include the interaction of CRP isoforms with LDL, and the impact on foam cell formation. Further studies are needed to determine exactly how the pCRP and mCRP configurations contribute to atherogenesis and CVD development, and how they could be targeted for effective CVD therapy.

## Abbreviations

CRP, C-reactive Protein; mCRP, monomeric CRP; pCRP, pentameric CRP; HCASMC, human coronary artery smooth muscle cell; HCAEC, human coronary artery endothelial cell; VSMC, vascular smooth muscle cell; HUVEC, human umbilical vein endothelial cell; HAEC, human aortic endothelial cell; oxLDL, oxidized LDL; ET-1, endothelin-1; LOX-1, Lectin-like oxidized LDL receptor-1; eNOS, endothelial NO-Synthase; EPC, endothelial progenitor cell; MCP-1, Monocyte chemoattractant protein-1; ROS, reactive oxygen species; AT1/2-R, Angiotensin II type 1/2 receptor; VV, vasa vasorum; MMP, matrix metalloproteinase.
